# Complete plastome sequence of *Mallotus japonicus* (Linn. f.) Müll. Arg. (Euphorbiaceae): a medicinal plant species endemic in East Asia

**DOI:** 10.1080/23802359.2021.1911707

**Published:** 2021-04-23

**Authors:** Wan-Ping Wu, Xiao-Feng Zhang, Zhi-Xin Zhu, Hua-Feng Wang

**Affiliations:** College of Tropical Crops, Hainan Key Laboratory for Sustainable Utilization of Tropical Bioresources, Hainan University, Haikou, China

**Keywords:** *Mallotus japonicas*, Euphorbiaceae, plastome, genome structure, phylogenetic

## Abstract

*Mallotus japonicus* is a shrub species in the family of Euphorbiaceae. The study of plastome would be helpful for its phylogenetic study and species identification. The total length of complete plastome for *Mallotus japonicus* is of 164,912 bp, with typical part-four structure and gene content of angiosperm plastome, including two inverted repeat (IR) regions of 27,829 bp, a large single-copy (LSC) region of 90,319 bp, and a small single-copy (SSC) region of 18,935 bp. The plastome contains 125 genes, consisting of 80 unique protein-coding genes, 31 unique tRNA gene, four unique rRNA genes (5S rRNA, 4.5S rRNA, 16S rRNA, and 23S rRNA), and five pseudogenes. The overall G/C content in the plastome of *Mallotus japonicus* is 40.2%. The phylogenetic analysis indicates that *M. japonicus* is closer to *M. peltatus* than other species in this study. The complete plastome sequence is conducive to the exploitation and utilization of Euphorbiaceae resources and the phylogenetic study in future.

*Mallotus japonicus* is a shrub species in the family of Euphorbiaceae, occurring in Taiwan, Zhejiang and Jiangsu province of China, and it is also distributed in Korea and Japan. *Mallotus japonicus* could be used as Chinese medicine (Satomi et al. 2001). Therefore, the study of plastome of *Mallotus japonicus* is of great significance to its medicinal use exploit and utilize and its systematic position. However, the plastome structure of *Mallotus japonicus* has not been well understood. In this report, we describe the complete plastome sequence of *Mallotus japonicus* (GenBank accession number: MW244068, this study), aiming at promoting the protection of germplasm resources and providing useful genomic resources. The samples of this study are collected from Ruili City, Yunnan Province (100.25°E, 26.86°N). A voucher specimen (voucher code, RL0046) and its DNA were deposited in the Herbarium of the Institute of Herbarium of China National GenBank (code of herbarium: HCNGB).

We analyzed the genomic DNA from *Mallotus japonicus* for quality and quantity with an Agilent BioAnalyzer 2100 (UCDAVIS Genome Center, Davis, CA). We shared about 0.8 μg of DNA and used it to prepare paired-end libraries with 200–400 bp insert size. We sequenced *Mallotus japonicus* with the BGISEQ-500 platform at BGI (Shenzhen, China), producing about eight Gb high quality sample with 100 bp paired-end reads. We trimmed raw reads with SOAPfilter_v2.2 (BGI, Shenzhen, China) using the following criteria: first, reads with >10% N’s; second, reads with >40% low quality bases (quality score <10); third, reads contaminated by adaptor sequence and produced by PCR duplication. About 6 Gb clean data were assembled against the plastome of *Ricinus communis* (GenBank accession number: JF937588.1, Rivarola et al. 2011) using MITO bim v1.8 (Le-Petit-Quevilly, France, Hahn et al. 2013). The plastome was annotated using Geneious R11.0.4 (Biomatters Ltd., Auckland, New Zealand) against the plastome of *Manihot esculenta* (GenBank accession number: NC010433.1).

The experimental results showed that the plastome length of *Mallotus japonicus* is 164,912 bp, with the typical quadrilateral structure characteristics of angiosperms, containing two inverted repeats (IRs) of 27,829 bp, a large single-copy (LSC) region of 90,319 bp, and a small single-copy (SSC) region of 18,935 bp. The plastome contains 125 genes, consisting of 80 unique protein coding genes (six of which are duplicated in the IR), 31 unique tRNA genes (seven of which are duplicated in the IR), and four unique rRNA genes (5S rRNA, 4.5S rRNA, 16S rRNA, and 23S rRNA). There are five pseudogenes in these genes. The overall G/C content in the plastome of *Mallotus japonicus* is 40.2%, and the corresponding values of the LSC, SSC, and IR region were 33.1%, 29.5%, and 41.5%, respectively.

The best-fitting model of nucleotide substitution for the plastome was determined by the Akaike information criterion (AIC) in jModelTest v2.1.7 (Santorum et al. 2014). We used RAxML (Stamatakis 2006) with 1000 bootstraps under the GTRGAMMAI substitution model to reconstruct a maximum-likelihood (ML) phylogeny of six published complete plastomes of Euphorbiaceae, using *Parastemon urophyllus* NC030517.1, *Maranthes glabra* NC030578.1 and *Exellodendron barbatum* NC030558.1 as outgroups. By constructing phylogenetic relationship, we find that *M. japonicus* and *M. peltatus* have a closer phylogenetic relationship than other taxa within Euphorbiaceae (Figure 1). The knowledge of plastome structure in *Mallotus japonicus* is beneficial to the exploitation and utilization of Euphorbiaceae resources, and also to the development of conservation genetics and phylogenetic study of Euphorbiaceae.

**Figure 1.**
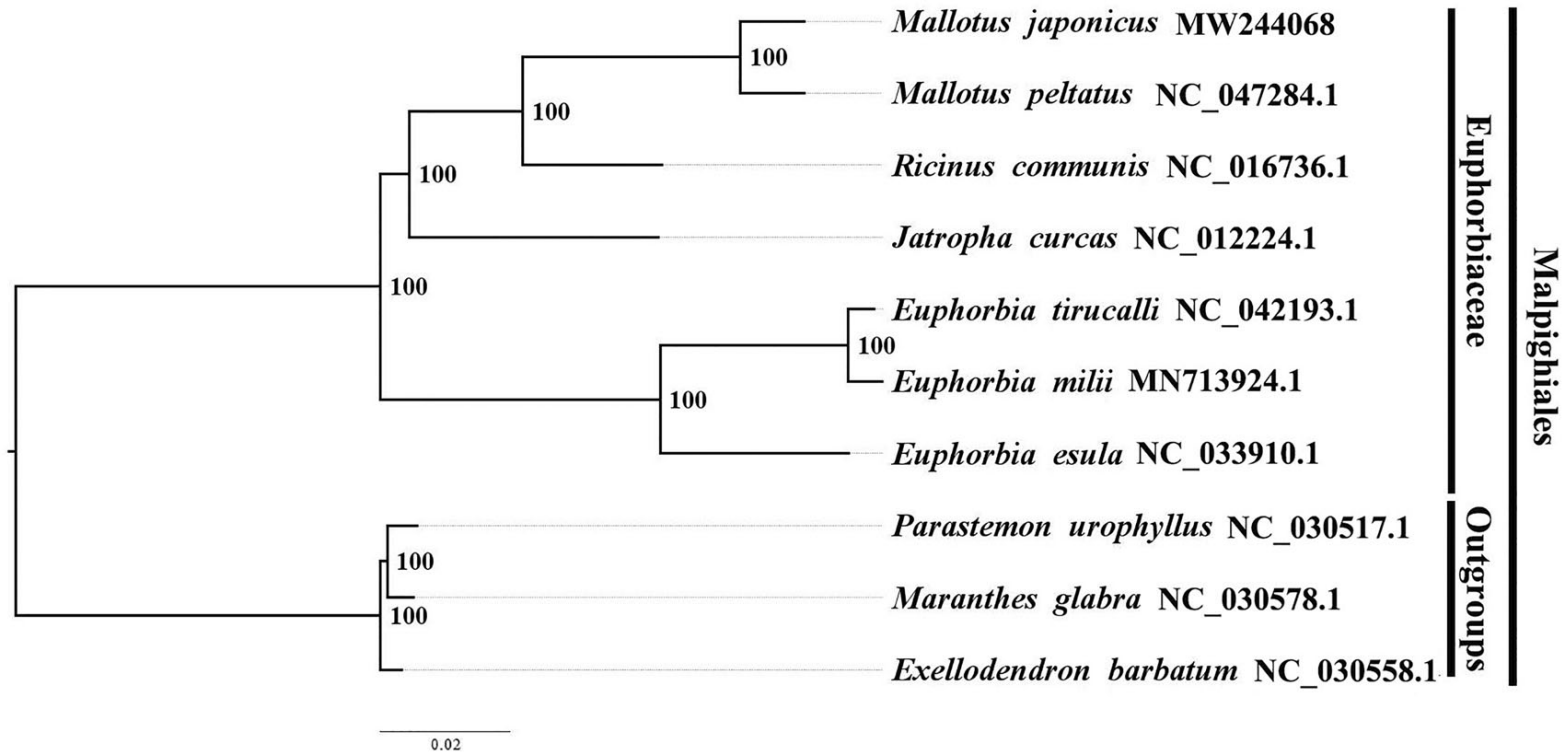
The maximum-likelihood (ML) phylogeny recovered from 10 complete plastome sequences by RAxML. Accession numbers: *Mallotus japonicus* (GenBank accession number, MW244068, this study), *Mallotus peltatus*, NC047284.1; *Ricinus communis*, NC016736.1; *Jatropha curcas*, NC012224.1; *Euphorbia tirucalli*, NC042193.1; *Euphorbia milii*, MN713924.1; *Euphorbia esula*, NC033910.1. Outgroups: *Parastemon urophyllus*, NC030517.1; *Maranthes glabra*, NC030578.1; *Exellodendron barbatum*, NC030558.1.

## Data Availability

The genome sequence data that support the findings of this study are openly available in GenBank of NCBI at https://www.ncbi.nlm.nih.gov/ under the accession no. MW244068. The associated BioProject, SRA, and Bio-Sample numbers are PRJNA 438407, SRS3260442, and SAMN08770363, respectively.
